# Emergency Pancreatoduodenectomy for Ampullary Cancer Post-Iatrogenic Duodenal Perforation: No Option but to Strike

**DOI:** 10.7759/cureus.11384

**Published:** 2020-11-08

**Authors:** Vaibhav K Varshney, Raghav Nayar, Kelu S Sreesanth, Subhash Soni, Bharti Varshney

**Affiliations:** 1 Surgical Gastroenterology, All India Institute of Medical Sciences, Jodhpur, Jodhpur, IND; 2 Pathology, All India Institute of Medical Sciences, Jodhpur, Jodhpur, IND

**Keywords:** endoscopic perforation, emergency, hepato biliary cancers, whipple's procedure, endoscopic retrograde cholangiopancreatography (ercp)

## Abstract

Endoscopic retrograde cholangiography related duodenal perforation is an infrequent complication and associated with significant morbidity. The management of such perforations, especially in the setting of malignancy, is not standardized given the paucity of literature.

We encountered a patient who was diagnosed with periampullary carcinoma and had a perforation in the duodenum during endoscopy. Emergency pancreatoduodenectomy (EPD) was performed considering it to be a resectable disease with minimal contamination. He had a prolonged hospital course due to surgical site infection and hepaticojejunostomy leak, however, which was managed successfully. At one year follow up, he is healthy with no evidence of recurrence.

We conclude that EPD can be attempted for selected iatrogenic duodenal perforations with co-existent resectable malignancy in a stable patient. It may help to avoid the morbidity of a second surgery in the setting of a distorted anatomy and simultaneously preventing the probable upstaging of disease due to peritoneal seedling.

## Introduction

Emergency pancreatoduodenectomy (EPD) has been carried out mainly for pancreatic trauma, bleeding aneurysms, iatrogenic duodenal perforations, and hemorrhage within a pseudocyst and has proven to be lifesaving in such instances. The mortality rates for EPD varies from 8-40% [1,2]. However, given the rarity of this surgery, there is very little published data regarding the same. Moreover, there is no defined protocol for the management of endoscopic perforations (EP) in the setting of malignancy and the management is primarily based on the treating physicians/surgeon's experience and skills. Here we describe a case of a patient with periampullary carcinoma (PACA) who had an endoscopy-related duodenal perforation which was recognized during the procedure and an EPD was performed.

## Case presentation

A 53-year-old male presented with complaints of painless progressive jaundice with cholestatic features and intermittent episodes of fever associated with anorexia and weight loss for one month. He had no history of prodromal symptoms, malaise, hematemesis, or melena. His liver function tests were as follows - Total bilirubin 8.4mg/dl, direct bilirubin 5.2 mg/dl, alkaline phosphatase -1031 IU/L, Aspartate transaminase-118 U/L, and Alanine transaminase-153 U/L.

**Figure 1:**
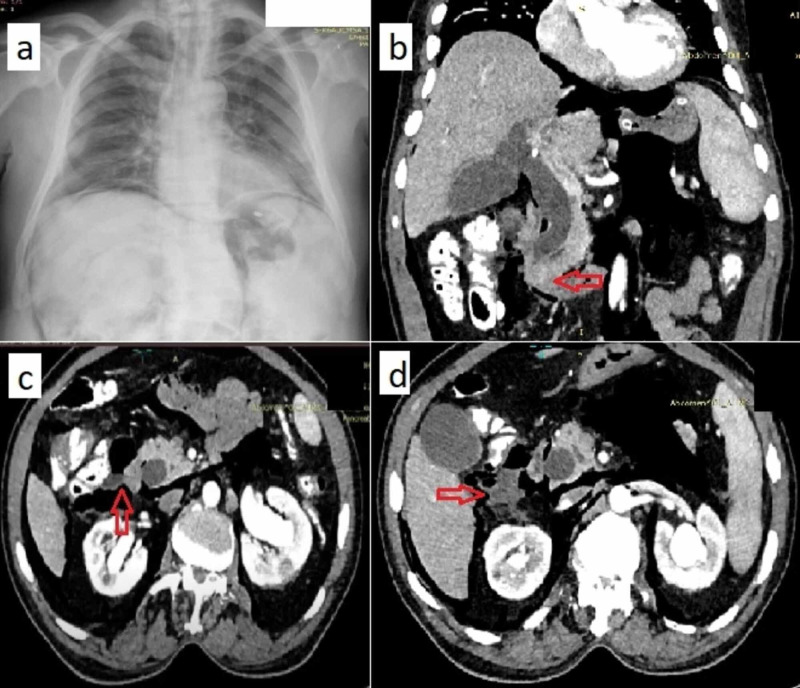
Chest radiograph and CECT Abdomen (a) Chest radiograph showing subcutaneous emphysema as well as pneumo-peritoneum and retroperitoneal free air; (b) CECT whole abdomen (coronal view) showing heterogeneously enhancing lesion in the periampullary region (red arrow) and dilated CBD; (c) and (d) CECT whole abdomen (axial views) showing perforation in the lateral wall of the duodenum with adjoining collection and pneumoperitoneum. CECT- Contrast-enhanced computed tomography

Ultrasound of the abdomen showed dilated intrahepatic biliary radicles and common bile duct (CBD). Endoscopic retrograde cholangiography (ERC) and stenting was planned in view of raised bilirubin levels with a history of fever. During the procedure, a periampullary growth was noted and before biopsy could be performed, the endoscopist noted an iatrogenic perforation in the second part of the duodenum. The procedure was abandoned and urgent surgical gastroenterology consultation was sought. Following the procedure, the patient was anxious with tachycardia, however, other vital parameters were within normal limits. His abdomen was distended with guarding noted in right hypochondrium and lumbar region. Subcutaneous emphysema was noted extending to the chest bilaterally (Figure 1a). Contrast-Enhanced Computed Tomography (CECT) abdomen was performed which showed heterogeneously enhancing soft tissue density lesion in the periampullary region with dilated CBD suggestive of PACA with no distant metastasis (Figure 1b). Extensive pneumoperitoneum with retroperitoneal extension and a collection of fluid with discontinuity in the lateral wall of the second part of the duodenum (D2) was noted (Figure 1c-1d). He was resuscitated and underwent an emergency laparotomy.

**Figure 2:**
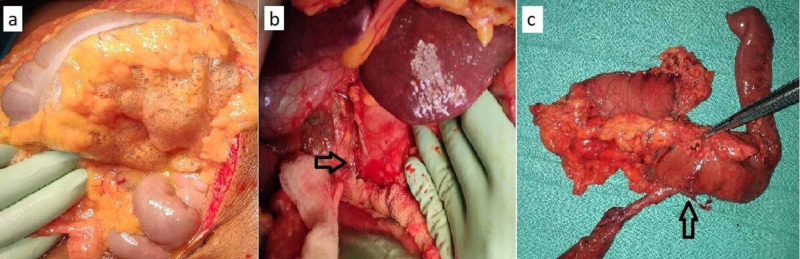
Intra-operative images (a) Showing air in the transverse mesocolon; (b) Black arrow showing perforation in the lateral wall of the duodenum; (c) Resected specimen of Pancreatoduodenectomy with perforation in the lateral wall of duodenum (black arrow).

Explorative laparotomy was performed within four hours following the perforation. Intraoperatively, contamination of the right para duodenal space and mesocolon was seen with ~1 x 1 cm perforation seen on the lateral aspect of the second portion of the duodenum (Figure 2a-2c). CBD was dilated (2.1 cm) with a 1 x 1 cm mass lesion noted in the periampullary region and the pancreas was soft in consistency with ~3 mm duct diameter. He underwent classical pancreatoduodenectomy (PD) with pancreaticojejunostomy (PJ) done using modified Blumgart’s technique. Feeding jejunostomy (FJ) was constructed 45 cm distal to gastrojejunostomy. Extensive peritoneal lavage was performed and abdominal drains were placed.

He was transferred to the Intensive Care Unit (ICU), where he was extubated on postoperative day (POD) one, and the inotropic support was gradually weaned off. Feeding through jejunostomy tube was commenced on POD two and oral feeds were started on POD three which he tolerated well. An amylase level from the abdominal drains on POD three was normal and the drains were subsequently removed.

On POD eight, he developed surgical site infection (SSI) which progressed to deep space infection. The wound was managed with daily dressings and antibiotics were upgraded according to culture sensitivity reports of the wound. Further, he started having biliary discharge from the surgical site for which CECT abdomen was done which suggested a hepaticojejunostomy leak without any obvious collection. A wound manager was applied on the midline wound and daily irrigation was done. On POD-16, he developed septic encephalopathy for which he had to be intubated and monitored in the ICU and later underwent tracheostomy on POD-22. He subsequently improved and enteral nutrition was continued through a jejunostomy tube. As his nutritional status improved, the bilious discharge from the wound gradually decreased and the wound showed signs of healing. Later, he was restarted on oral feeds on POD-30 and his tracheostomy was downsized and subsequently removed. Finally, his wound completely healed by secondary intention and he was discharged on POD-45 in a stable condition.

His pathology report of the lesion was suggestive of ampullary adenocarcinoma (stage pT1N0Mx) with all resection margins free of tumor. At one year follow-up, he is doing well without any evidence of recurrence.

## Discussion

Iatrogenic perforation during ERC varies from 0.01 to 2.1% and is associated with considerable morbidity [3,4]. Stapfer et al have classified iatrogenic endoscopic perforations into four types depending on the radiological association [5]. This helped to predict likely early surgical candidates with type I perforations (lateral duodenal wall) tending to be larger and hence requiring surgical interventions more frequently. CECT abdomen with oral contrast is usually helpful in confirmation by demonstrating contrast extravasation, free retroperitoneal air, or massive subcutaneous emphysema. The delay in surgical management in the context of scope induced duodenal perforations are associated with a higher risk of mortality [5].

Almost 15-40% of iatrogenic perforations occur in the presence of a co-existent malignancy, however, there is a lack of a defined protocol for their management due to scarcity of published literature [3,6]. The treatment in such cases is based on an individualized patient-centered approach. Medical management along with complete bowel rest, proton pump inhibitor therapy, parenteral antibiotics, and somatostatin analogs is preferred in stable patients without any signs of peritonitis or contrast extravasation on imaging. Recently, endoscopic methods have been proposed to treat these inadvertent EP including the use of endoscopic clips and fibrin glue, however, they are being used at selected centers and are contraindicated in large perforations and unstable patients [4].

Surgical intervention is warranted in patients with overt signs of peritonitis, large intra/retro-peritoneal air, hemodynamic instability, or failure of medical management. Surgical management primarily involves draining intra and retroperitoneal fluid collections, primary repair of the perforation with or without duodenal diversion [7].

Emergency Pancreatoduodenectomy (EPD) has been employed for managing iatrogenic duodenal or bile duct perforation in the background of malignancy. On reviewing the literature, only three cases have been reported where successful early (within 24 hours) EPD was performed for pancreatobiliary malignancy in which a scope induced perforation occurred [4,8]. Our case has added to this category where the patient had a resectable PACA, an iatrogenic perforation occurred and EPD was performed (Table 1).

**Table 1:** Detail of reports in which Emergency Pancreatoduodenectomy was performed for iatrogenic perforation in patients of malignancy #-Endoscopic Retrograde Cholangiography; ^ Esophagogastroduodenosope; *Hospital stay in days; NM-Not mentioned.

No.	Author	Age	Sex	Intervention	Perforation site	Final diagnosis	HS*
1	Tavusbay et al [4]	NM	F	NM	Duodenum	Ampullary cancer	NM
2	Standop et al [8]	77y	M	ERC^#^ stenting	Distal bile duct	Papillary cancer	56
3	Standop et al [8]	67y	F	EGD^^^ induced	Duodenum	Neuroendocrine cancer	27
4	Our case (Varshney et al)	53y	M	ERC^#^ stenting	Duodenum	Ampullary Adenocarcinoma	45

Early definitive surgery may prove beneficial in such cases but given the limited literature describing such cases, it is difficult to make concrete conclusions. The therapeutic benefit may be speculated since perforation in the setting of malignancy may upstage the disease with the risk of tumor seeding in the peritoneal cavity and disease progression. The initial hospital stay may be prolonged in such a setting but it avoids the morbidity of a second definite procedure in which altered anatomy with dense adhesions might make the pancreatoduodenectomy more complex. However, one must pay attention to the early identification, resuscitation, and prompt mobilization of these patients to the operation theatre as delays may contribute to dismal outcomes. Another important aspect is postoperative morbidity, as such patients are liable to have wound-related (16%) and anastomotic leak related (33%) complications [2,9]. In the present case, similar complications were seen, however, the favorable tumor biology, early-stage, and prompt post-operative management aided the patient with a good recovery.

Alternative procedures that might have been tried in the emergency setting were a primary repair of the perforation with pyloric exclusion with/without biliary drainage but the rates of mortality and post-operative duodenal leaks in such cases are extremely high [7]. Further, this would have deferred the definitive procedure.

Mortality rates in EP vary from 8-40% with considerable variability depending on the primary pathology, timely surgical intervention, and whether the surgery was therapeutic or diagnostic [1-3,5,9]. Delay in treatment of more than 24 hours after the perforation can result in doubling of mortality rates. The management of EP is not standardized in view of the low incidence of this complication and the heterogeneity of the patient population experiencing this procedure with respect to the site, type, presentation, and comorbidities [7].

## Conclusions

In conclusion, EPD can be considered selectively in iatrogenic duodenal perforations of patients with resectable periampullary tumors, stable hemodynamics, and an experienced surgeon/center. It is important to consider this in a selective cohort of patients only, given the high postoperative morbidity and mortality rates. Early surgery before the onset of sepsis will have favorable outcomes, however, the real challenge lies in anticipation and early identification of patients requiring surgery.

However, in unstable patients with diagnostic uncertainty, primary closure of duodenum with or without biliary drainage with feeding jejunostomy would be a safer option and definitive procedure should be deferred for the future.
